# Association of S2 Alar–Iliac Versus S1-Only Fixation with Spinopelvic Mobility and Acetabular Component Orientation in Total Hip Arthroplasty: A Standing–Sitting EOS Study

**DOI:** 10.3390/jcm15218210

**Published:** 2026-10-22

**Authors:** Burak Abay, Meric Enercan, Azmi Hamzaoglu

**Affiliations:** 1 Department of Orthopedics and Traumatology, Demiroglu Bilim University, 34381 Istanbul, Turkey; 2 Scoliosis and Spine Center—Istanbul, Istanbul Florence Nightingale Hospital, 34381 Istanbul, Turkey

**Keywords:** total hip arthroplasty, spinopelvic fixation, S2 alar–iliac screw, sacral slope, pelvic–femoral angle, acetabular component orientation, EOS imaging, hip–spine relationship

## Abstract

**Background:** S2 alar–iliac (S2AI) fixation crosses the sacroiliac joint, unlike fixation terminating at S1, and may be associated with reduced spinopelvic mobility in patients undergoing total hip arthroplasty (THA). We compared spinopelvic mobility, femoropelvic reorientation, acetabular component orientation, and hip-specific function between S2AI and S1-only fixation. **Methods:** This retrospective comparative cohort study included 60 patients undergoing THA after adult spinal fusion with S2AI (*n* = 30) or S1-only (*n* = 30) fixation. Standing and sitting EOS imaging at 1 year assessed changes in sacral slope (ΔSS) and pelvic–femoral angle (ΔPFA). Immediate postoperative CT measured static acetabular component orientation. Hip-specific function was assessed using the Hip Disability and Osteoarthritis Outcome Score (HOOS). Multivariable regression evaluated associations with ΔSS and HOOS. **Results:** Spinopelvic stiffness (ΔSS < 10°) was more frequent with S2AI fixation (66.7% vs. 13.3%; *p* < 0.001). Compared with S1-only fixation, S2AI was associated with lower ΔSS (7.7 ± 4.8° vs. 18.1 ± 7.0°; *p* < 0.001) and greater ΔPFA (68.8 ± 11.7° vs. 58.7 ± 10.8°; *p* = 0.001). Static cup orientation was similar. One-year HOOS was lower with S2AI fixation (79 [68–86] vs. 86 [72–92]; *p* = 0.01), although ΔHOOS did not differ significantly (*p* = 0.07). After adjustment, S2AI fixation remained associated with lower ΔSS (β = −7.67; *p* < 0.001), while greater ΔSS was associated with higher HOOS (β = 3.42 per 5°; *p* = 0.003). **Conclusions:** S2AI fixation was associated with reduced spinopelvic mobility and greater femoropelvic reorientation despite similar static cup orientation. Residual confounding by spinal construct complexity precludes causal inference.

## 1. Introduction

Total hip arthroplasty (THA) performed after prior lumbar or lumbopelvic fusion is associated with an increased risk of instability and revision [1]. This elevated risk has been linked to altered spinopelvic mechanics and reduced postural adaptability [2,3]. During normal standing-to-sitting transition, the pelvis rotates posteriorly, sacral slope decreases, and lumbar lordosis is reduced, resulting in increased functional acetabular anteversion. In patients with a stiff or fused spine, this adaptive pelvic rotation may be restricted, potentially altering functional acetabular orientation and increasing the risk of impingement or instability [3,4]. Consequently, assessment of the dynamic hip–spine relationship has become increasingly relevant in THA planning, particularly in patients with previous spinal fusion.

Spinopelvic mobility is quantified as the standing-to-sitting change in sacral slope (ΔSS), which reflects the ability of the pelvis to adapt during postural transition [3,5]. With preserved mobility, posterior pelvic rotation during sitting increases functional acetabular anteversion and contributes to the overall motion required to achieve a seated posture. When this lumbopelvic contribution is restricted, greater compensatory motion may be required at the hip [5,6,7]. Accordingly, similar static acetabular component orientation may result in different functional biomechanics depending on the patient’s residual spinopelvic mobility. Previous studies have demonstrated substantial variation in functional cup orientation across spinopelvic mobility patterns and have emphasized the limitations of relying on static component orientation alone [6,7,8]. This hip–pelvis relationship is reciprocal: after THA, pelvic and lumbar mobility decreases while hip mobility increases, with pelvic motion in part driven by the hip [9]. Excessive compensatory hip motion may increase the mechanical demands across the hip, while longer spinal fusion has been associated with accelerated hip joint-space narrowing [10].

Among sacropelvic fixation techniques, S2 alar–iliac (S2AI) screws are widely used in adult spinal deformity surgery because they provide robust distal fixation with a lower implant profile and fewer hardware-related complications than traditional iliac screws [11]. Unlike constructs terminating at S1, S2AI screws traverse the sacroiliac joint and extend fixation into the ilium [12]. This distinction may be biomechanically relevant to subsequent THA because the sacroiliac joint contributes to the motion of the lumbopelvic complex [13]. Experimental and finite-element studies suggest that lumbopelvic constructs extending across the sacroiliac joint may alter load transfer and increase compensatory motion and mechanical demand at the hip [14,15]. Clinical observations in adult spinal deformity populations further suggest an important role for hip compensation after S2AI fixation [16]. However, whether S2AI fixation is associated with a different residual spinopelvic mobility pattern than S1-only fixation in patients subsequently undergoing THA, and whether such differences are related to hip-specific outcomes, remains unclear.

Therefore, the aim of this study was to compare patients undergoing THA after adult spinal deformity reconstruction with S2AI versus S1-only fixation by integrating static CT-based acetabular component orientation, standing-to-sitting spinopelvic mobility, femoropelvic reorientation, and hip-specific outcomes. We hypothesized that S2AI fixation would be associated with reduced residual spinopelvic mobility and greater femoropelvic reorientation despite comparable static acetabular component orientation, and that reduced residual spinopelvic mobility would be associated with poorer hip-specific outcomes after THA.

## 2. Materials and Methods

### 2.1. Study Design, Setting and Patient Selection

This retrospective, comparative cohort study was conducted at a single tertiary referral centre and reported in accordance with the STROBE guidelines for cohort studies. We reviewed consecutive patients who had undergone spinal fusion for adult spinal deformity (ASD) and subsequently underwent primary THA between January 2015 and January 2025. Patients were classified according to the distal fixation as S2 alar–iliac (S2AI) or S1-only fixation group.

Patients were eligible if spinal fusion had been performed at least 1 year before THA, clinical and radiographic follow-up after THA was at least 1 year, and standing and relaxed-sitting EOS imaging was available at the 1-year postoperative assessment. EOS imaging system (EOS Imaging, Paris, France) was available throughout the study period. Four patients underwent staged bilateral THA (3 S1-only, 1 S2AI), with the second procedure performed 6 months after the first; only the first-operated hip was analyzed, and PFA was measured on that hip. Exclusion criteria were incomplete follow-up, absence of the required 1-year EOS examination, death before the 1-year assessment, subsequent spinal revision before this assessment, or prior spinal surgery for non-deformity indications. Clinical and radiographic follow-up data were reviewed through January 2026.

Distal fixation strategy was determined by the treating spine surgeon according to deformity severity, fixation requirements, and patient-specific factors. The number of fused segments was defined as the number of fused motion segments from the upper instrumented vertebra (UIV) to S1 (L5–S1 counted as one segment); pelvic fixation was not counted as an additional segment. A long construct was defined as fusion involving ≥5 segments. History of revision spinal fusion before THA, number of fused segments, upper instrumented vertebral level, L5–S1 pseudarthrosis or distal failure, major corrective osteotomy, and DXA-confirmed osteoporosis were recorded as indicators of spinal surgical complexity. Sagittal alignment was assessed before spinal reconstruction, after spinal reconstruction but before THA, and at 1 year after THA. Baseline spinal surgical characteristics and pre- and post-spinal-fusion alignment parameters are presented in Table 1.

The study was approved by the Institutional Review Board of Demiroglu Bilim University (Approval No: 2026-01-06; 28 January 2026) and conducted in accordance with the Declaration of Helsinki. Clinical and imaging data were collected during routine care; after ethics approval, written informed consent for participation in this study and for publication of anonymized data was obtained from all included patients.

### 2.2. Study Groups and Surgical Technique

The S2AI group (*n* = 30) underwent bilateral S2AI fixation, with screws inserted between the S1 and S2 dorsal foramina and directed across the sacroiliac joint toward the anterior inferior iliac spine, superior to the greater sciatic notch [12,17]. In the S1-only group (*n* = 30), fixation terminated at S1 and did not cross the sacroiliac joint. The distal fixation techniques are illustrated schematically in Supplementary Figure S1.

All THAs were performed by a single senior surgeon, predominantly through a direct lateral approach. Uncemented components were used in all patients. Dual-mobility implants (32 mm inner head) were used at the surgeon’s discretion in patients considered at higher risk of dislocation and were analyzed as a separate category. Acetabular component orientation was not individualized according to preoperative spinopelvic mobility or distal spinal fixation type. All patients followed the same institutional postoperative rehabilitation protocol.

### 2.3. Radiographic and Functional Evaluation

Immediate postoperative multidetector CT scanner (Siemens Healthineers, Forchheim, Germany) was used to assess the static acetabular component anteversion and inclination relative to the anterior pelvic plane.

Standing and relaxed-sitting biplanar EOS images were obtained at 1 year after THA. Sacral slope (SS), pelvic tilt (PT), lumbar lordosis (LL), and pelvic–femoral angle (PFA) were measured in both positions. All postural changes were calculated as standing minus sitting. Spinopelvic mobility was quantified as ΔSS, with stiffness defined as ΔSS < 10° [3,9]. ΔPFA represented standing-to-sitting femoropelvic reorientation and was not interpreted as intrinsic or maximum hip range of motion. Relaxed sitting was performed without standardization of seat height, foot support, trunk posture, or thigh position; patients were asked to sit comfortably in their usual manner. Representative measurements of SS, PT, and PFA in standing and relaxed-sitting positions are illustrated in Supplementary Figure S2. For the regression analyses, PI–LL mismatch was measured after spinal reconstruction but before THA, whereas postoperative SVA was measured on standing EOS radiographs obtained 1 year after THA.

Radiographic measurements were performed independently by two orthopedic surgeons (one arthroplasty surgeon [B.A.] and one spine surgeon [M.E.]). Because fixation was visible on EOS imaging, observers could not be blinded to fixation type but had no access to clinical outcomes or patient-reported scores. SS, PT, LL, and PFA were measured on standing and sitting EOS images in all 60 patients, and the first observer repeated all measurements after 4 weeks, blinded to the initial results. Reliability was quantified using the intraclass correlation coefficient (ICC; two-way random-effects, absolute agreement, and single measures) with 95% confidence intervals. Mean interobserver and intraobserver ICCs across parameters were 0.93 (range 0.92–0.95) and 0.95 (range 0.94–0.96), respectively; parameter-specific values are given in Supplementary Table S1.

### 2.4. Evaluation of Patient-Reported Outcomes

Hip-specific outcomes were assessed preoperatively and at 1 year after THA using the Turkish full version of the 40-item Hip Disability and Osteoarthritis Outcome Score (HOOS) [18]. Each item is scored from 0 (no problem) to 4 (extreme problem). The total HOOS was calculated as defined in the Turkish validation study [18]: all 40 items were summed and transformed to a 0–100 scale (100 = no symptoms; higher scores indicating better hip-related health). In that study, the total score showed Cronbach’s α = 0.98 and test–retest ICC = 0.84 (95% CI 0.74–0.90) [18]. Because the total score is the sum of all items, the five domains contribute in proportion to their number of items (symptoms 5, pain 10, activities of daily living [ADL] 17, sport and recreation 4, and quality of life 4); ADL therefore accounts for 17 of 40 items. Complete HOOS data were available, and no imputation was required. The five domain scores are reported descriptively in Supplementary Table S2.

The validated Turkish Oswestry Disability Index (ODI) was assessed at the same time points as a secondary measure of spine-related disability [19,20]; scores range from 0 to 100, with higher scores indicating greater disability.

### 2.5. Clinical Events

THA-related clinical events included dislocation, symptomatic anterior impingement, and revision surgery. Dislocation was defined as radiographically confirmed loss of articulation requiring closed or open reduction. Symptomatic anterior impingement was defined as anterior hip or groin pain reproducibly provoked by active or resisted hip flexion and clinically considered consistent with prosthetic impingement. Alternative causes were excluded by clinical assessment, radiographic evaluation for loosening or periprosthetic fracture, and hematologic inflammatory markers for periprosthetic joint infection. Revision was defined as reoperation involving exchange, removal, or addition of one or more prosthetic components.

### 2.6. Statistical Analysis

Continuous radiographic parameters showed no significant departure from normality on Shapiro–Wilk testing (all *p* > 0.05) and were summarized as mean ± SD and compared using independent-samples t tests. Between-group differences are presented as mean differences (S1-only minus S2AI) with 95% confidence intervals. To address construct complexity, the ΔSS comparison was repeated in patients with long constructs only (≥5 fused segments). HOOSs were non-normally distributed and were summarized as median [IQR], with Mann–Whitney U tests for between-group comparisons and Wilcoxon signed-rank tests for within-group changes. ODI distributions and change scores showed no significant departure from normality (all *p* > 0.05) and were analyzed using independent-samples and paired t tests, respectively. Categorical variables were compared using the two-sided Fisher’s exact test; the three-category femoral head diameter distribution was compared using the Fisher–Freeman–Halton exact test.

Multivariable linear regression was used to evaluate factors associated with ΔSS (Model A) and 1-year HOOS (Model B). As a sensitivity analysis, Model A was refitted using pre-THA SVA instead of 1-year SVA. Six predictors were selected based on clinical relevance, potential confounding, and the prior literature rather than data-driven selection. Model assumptions were assessed separately for each model. Multicollinearity was low (VIF range: Model A, 1.27–2.45; Model B, 1.35–3.21). Residual and Q–Q plots showed no substantial violations of model assumptions. Breusch–Pagan tests did not indicate heteroscedasticity (Model A, *p* = 0.24; Model B, *p* = 0.16), and Cook’s distance was < 1 for all observations in both models. ΔSS was designated as the primary radiographic outcome in the protocol submitted for ethics approval (28 January 2026); because the study was retrospective, all clinical and imaging data had already been collected at that time, and the analysis plan was not prospectively registered. All tests were two-sided, with *p* < 0.05 considered significant. No adjustment for multiple comparisons was applied; apart from ΔSS, all other comparisons (including ΔPT, ΔPFA, patient-reported outcomes and complications) were considered secondary and exploratory, and their *p* values should be interpreted accordingly. Analyses were performed in R software (version 4.5.1; R Foundation for Statistical Computing, Vienna, Austria).

## 3. Results

### 3.1. Study Cohort

**Figure 1:**
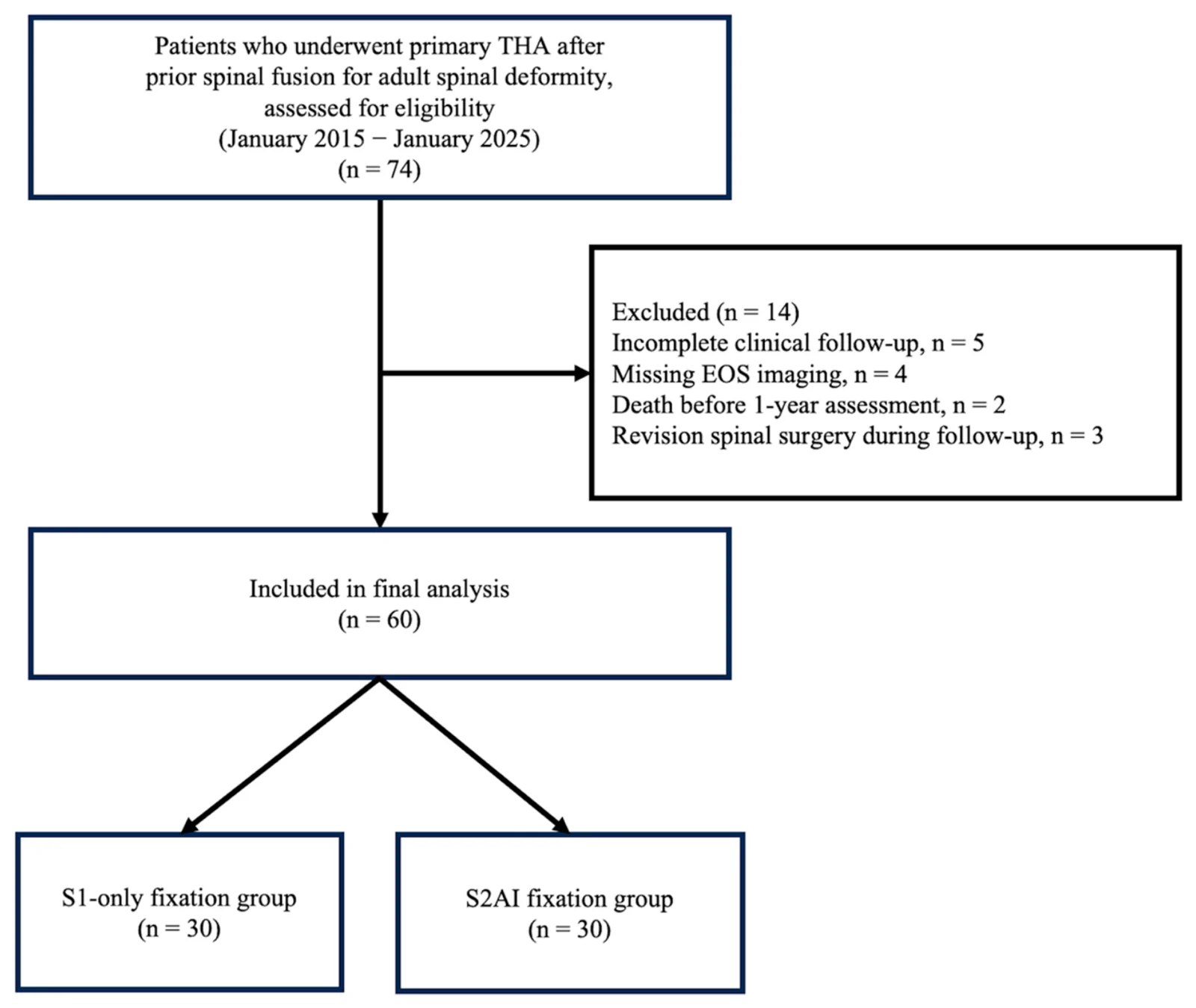
Flow diagram of patient selection.

Seventy-four potentially eligible patients were identified by retrospective screening. Fourteen were excluded (six S1-only and eight S2AI): incomplete follow-up (*n* = 5; one S1-only, four S2AI), unavailable 1-year EOS examination (*n* = 4; two S1-only, two S2AI), death before the 1-year assessment (*n* = 2; one per group), and subsequent spinal revision surgery before the standardized 1-year assessment (*n* = 3; two S1-only, one S2AI). No patient was excluded for prior spinal surgery for non-deformity indications. The final cohort comprised 60 patients (30 S1-only and 30 S2AI). Patient selection is summarized in Figure 1.

Age, sex, and body mass index were similar between groups. The S2AI group had more fused segments (8.7 ± 2.1 vs. 6.6 ± 2.6; *p* = 0.001). Revision spinal fusion before THA and UIV at T10 or above was numerically more frequent in the S2AI group, whereas long constructs were frequent in both groups (80.0% vs. 90.0%; *p* = 0.47). Pre- and post-spinal sagittal alignment parameters did not differ significantly between groups (Table 1).

### 3.2. THA Characteristics, Static Component Orientation, and Adverse Events

THA approach, femoral head-size distribution, and use of dual-mobility implants were similar between groups (Table 2). Static acetabular component orientation on immediate postoperative CT did not differ between groups: anteversion was 21.8 ± 5.0° in the S1-only group and 23.1 ± 5.4° in the S2AI group (*p* = 0.34), with similar inclination (*p* = 0.53).

Dislocation occurred in one patient in each group. Symptomatic anterior impingement occurred in one S1-only patient and four S2AI patients. Two patients in the S2AI group underwent revision THA for symptomatic anterior impingement. Both revisions occurred after the standardized 1-year EOS and HOOS assessments, at 16 and 15 months after the index THA, respectively, and both involved acetabular component revision with head and liner exchange. Given the small number of events, these findings are descriptive and hypothesis-generating.

### 3.3. Standing-to-Sitting Spinopelvic Mobility and Femoropelvic Reorientation

Standing EOS parameters were similar between groups (all *p* ≥ 0.37), whereas differences emerged in the relaxed-sitting position and in standing-to-sitting postural changes (Table 3). 

**Figure 2:**
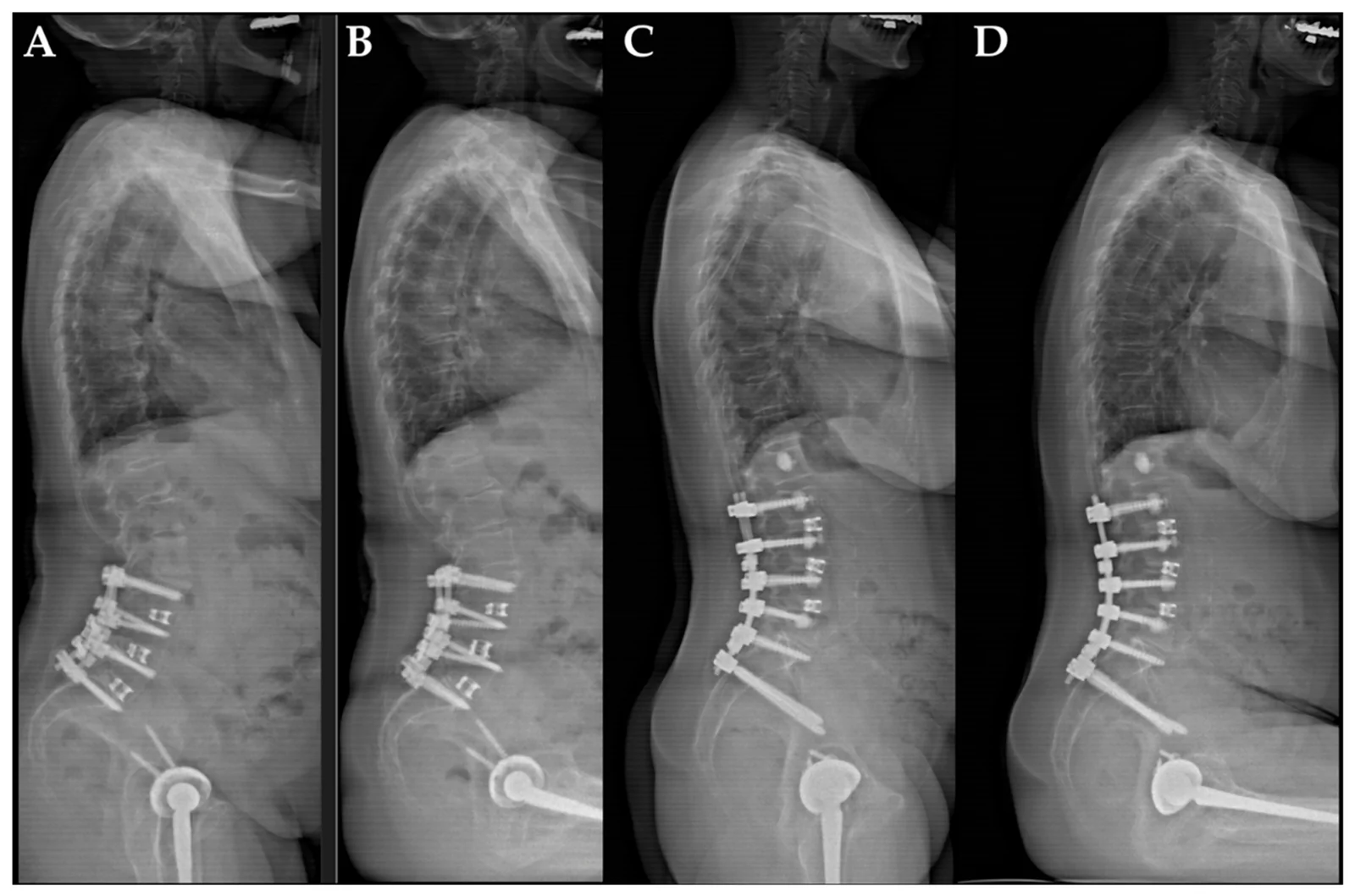
Representative lateral standing and sitting EOS radiographs illustrating differential residual spinopelvic mobility between fixation groups after total hip arthroplasty (THA). (**A**,**B**) S1-only fixation, representative patient: standing (**A**) and sitting (**B**) radiographs show marked posterior pelvic rotation between positions, consistent with preserved spinopelvic mobility. (**C**,**D**) S2AI fixation, representative patient: standing (**C**) and sitting (**D**) radiographs show comparatively little change in pelvic orientation between positions despite a well-positioned THA component, illustrating lower standing-to-sitting pelvic reorientation in this representative S2AI patient.

Spinopelvic stiffness (ΔSS < 10°) was present in 4 of 30 S1-only patients (13.3%) and 20 of 30 S2AI patients (66.7%); the distribution differed significantly (*p* < 0.001). Mean ΔSS was greater in the S1-only group (18.1 ± 7.0° vs. 7.7 ± 4.8°; mean difference 10.4°, 95% CI 7.3 to 13.5; *p* < 0.001), whereas ΔPFA was greater in the S2AI group (68.8 ± 11.7° vs. 58.7 ± 10.8°; mean difference 10.1°, 95% CI 4.4 to 15.8; *p* = 0.001), indicating greater standing-to-sitting femoropelvic reorientation. In a sensitivity analysis restricted to patients with long constructs (≥5 fused segments; *n* = 24 S1-only, *n* = 27 S2AI), ΔSS remained lower with S2AI fixation (7.5 ± 4.8° vs. 18.3 ± 7.1°; mean difference 10.7°, 95% CI 7.4 to 14.1; *p* < 0.001), and spinopelvic stiffness was more frequent (70.4% vs. 12.5%; *p* < 0.001). Representative standing and sitting radiographs from each group are shown in Figure 2.

### 3.4. Patient-Reported Outcomes

Preoperative HOOS was similar between groups (45 [41–58] vs. 47 [38–59]; *p* = 0.32). At 1 year, total HOOS was higher in the S1-only group than in the S2AI group (86 [72–92] vs. 79 [68–86]; Hodges–Lehmann difference 7.2 points, 95% CI 1.5 to 12.8; *p* = 0.01). The improvement from baseline did not differ significantly between groups (+37 [28–40] vs. +31 [21–39]; Hodges–Lehmann difference 6.1 points, 95% CI −0.4 to 12.5; *p* = 0.07). Both groups improved significantly from baseline (both *p* < 0.001). ODI did not differ between groups preoperatively, at 1 year, or in the magnitude of change (all *p* ≥ 0.39) (Table 4).

### 3.5. Multivariable Analysis

**Figure 3:**
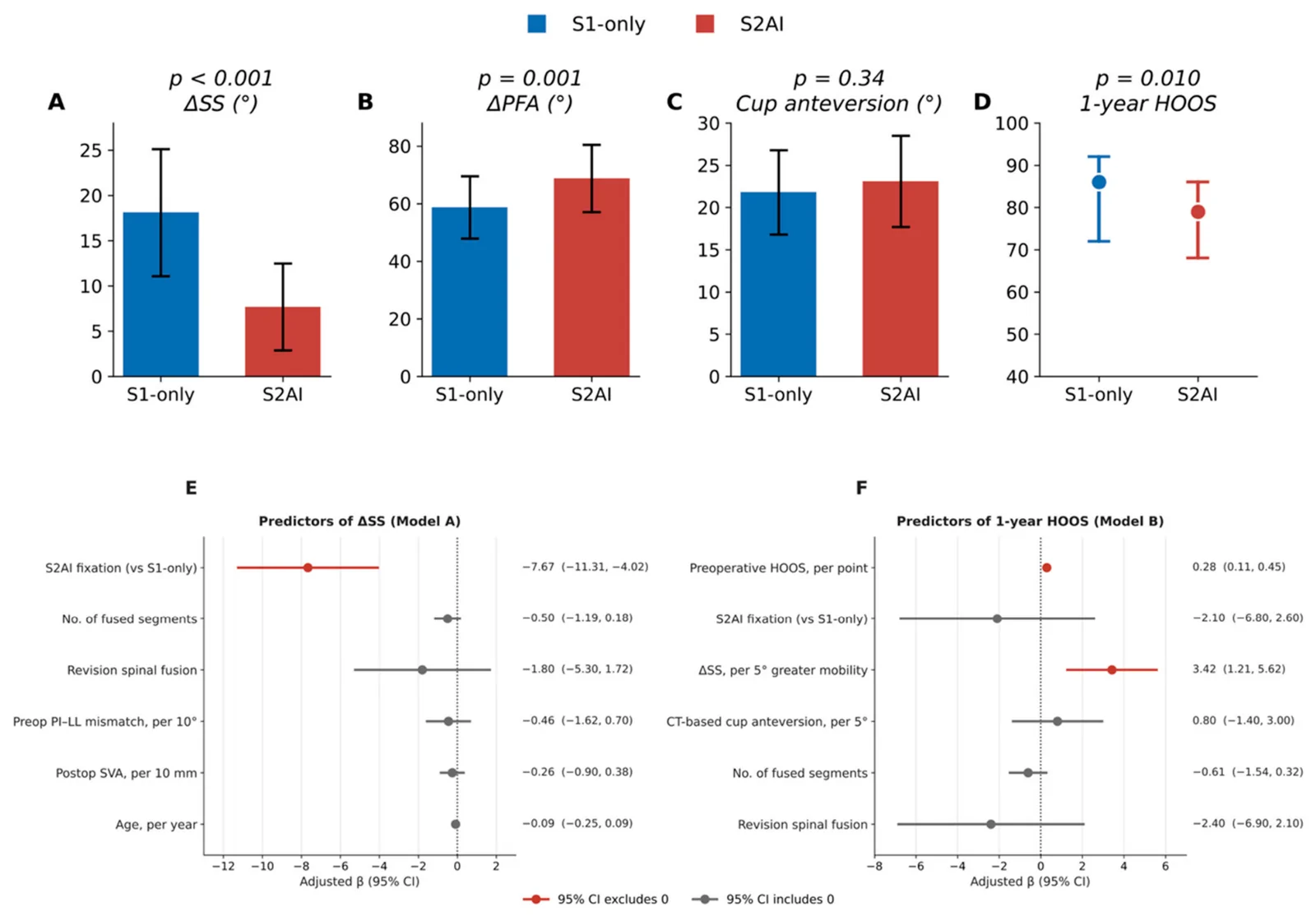
Group differences and multivariable predictors of spinopelvic mobility and hip function. (**A**–**C**) Between-group comparison of standing-to-sitting change in sacral slope (ΔSS), standing-to-sitting change in pelvic–femoral angle (ΔPFA), and CT-based cup anteversion, presented as mean ± SD; *p* values were obtained using independent-samples t tests. (**D**) One-year HOOS presented as median [IQR], with the between-group *p* value obtained using the Mann–Whitney U test. (**E**) Adjusted β coefficients (95% CI) for predictors of ΔSS (Model A). (**F**) Adjusted β coefficients (95% CI) for predictors of 1-year HOOS (Model B). Red markers indicate predictors whose 95% CI excludes zero; grey markers indicate predictors whose 95% CI includes zero. HOOS, Hip disability and Osteoarthritis Outcome Score; CT, computed tomography; PI–LL, pelvic incidence minus lumbar lordosis; SVA, sagittal vertical axis.

In Model A, S2AI fixation was associated with lower ΔSS after adjustment for measured covariates (β = −7.67, 95% CI −11.30 to −4.02; *p* < 0.001), whereas the number of fused segments was not significantly associated with ΔSS (β = −0.50; *p* = 0.16). Results were unchanged when pre-THA SVA replaced 1-year SVA (S2AI β = −7.67, 95% CI −11.30 to −4.02; *p* < 0.001). In Model B, greater ΔSS was associated with higher 1-year HOOS (β = 3.42 per 5° increase, 95% CI 1.21 to 5.62; *p* = 0.003), whereas S2AI fixation was not independently associated with HOOS (β = −2.10; *p* = 0.37). Full model estimates are presented in Table 5, and group differences and adjusted model coefficients are summarized graphically in Figure 3.

## 4. Discussion

### 4.1. Principal Findings

This study compared S2AI and S1-only fixation in patients who later underwent THA. Spinopelvic mobility was lower after S2AI fixation, with less than half of the standing-to-sitting change in sacral slope seen in the S1-only group (7.7° vs. 18.1°), and two-thirds of S2AI patients met the definition of spinopelvic stiffness compared with about one in seven of those in the S1-only group. Femoropelvic reorientation was correspondingly greater in the S2AI group. Static acetabular component orientation on CT was almost identical between groups, yet 1-year hip function was lower after S2AI fixation. Symptomatic impingement and revision were numerically more frequent in the S2AI group, whereas dislocation occurred at the same low rate in both groups; given the small number of events, these findings should be considered descriptive. In multivariable analysis, S2AI fixation remained associated with lower ΔSS after adjustment for measured covariates, whereas greater ΔSS was associated with better 1-year HOOS.

### 4.2. S2AI Fixation and Spinopelvic Mobility

The reduced ΔSS in the S2AI group is consistent with restricted standing-to-sitting pelvic reorientation in the presence of sacroiliac-joint-spanning fixation. In contrast, S1-only fixation does not directly immobilize the sacroiliac joint, while bilateral S2AI screws cross it and may influence the sacropelvic contribution to standing-to-sitting retroversion. On multivariable analysis, this association persisted after adjustment for the number of fused segments, revision status, and sagittal alignment (adjusted β = −7.67; 95% CI −11.30 to −4.02; *p* < 0.001), and the number of fused segments itself was not an independent predictor (*p* = 0.16). However, ΔSS reflects overall standing-to-sitting pelvic reorientation rather than motion specifically across the sacroiliac joint, and the greater construct complexity in the S2AI group means that residual confounding cannot be excluded.

Our findings agree with earlier reports: Ukai et al. showed that long fusion accelerates hip joint space narrowing relative to short or no fusion [10]; Kim et al. described a reciprocal fall in pelvic mobility alongside a rise in hip mobility after THA [9]; and Li et al. found that after S2AI fixation the hip becomes the main compensator, with limited hip compensatory capacity predicting more mechanical complications [16]. Finite-element work by Kozaki et al. corroborates the pattern, showing that sacroiliac joint spanning fixation increases hip contact stress and angular motion, with more rigid constructs producing the greatest effect [14,15].

### 4.3. Femoropelvic Reorientation and Its Association with Function

ΔPFA was greater in the S2AI group (68.8° vs. 58.7°; *p* = 0.001), but it mirrors rather than adds to the ΔSS finding: the between-group differences in ΔPFA and ΔSS were nearly identical (10.1° and 10.4°), suggesting that the femur ended up in a similar orientation in both groups and that the larger ΔPFA after S2AI fixation reflects the smaller pelvic reorientation. Because sitting posture was not standardized, ΔPFA should not be read as hip range of motion, and values from flexed-seated protocols are not directly comparable [21].

Greater ΔSS was associated with higher 1-year HOOS (adjusted β = 3.42 per 5° of mobility; *p* = 0.003), while S2AI fixation was not independently associated with HOOS after ΔSS and the other measured covariates were included in the model (β = −2.10; *p* = 0.37). Although the association between fixation type and HOOS was attenuated after inclusion of ΔSS, ΔSS and HOOS were assessed concurrently at the 1-year follow-up; therefore, this finding should not be interpreted as evidence of mediation. Static CT-based cup anteversion was also not independently associated with HOOS in the same model (*p* = 0.47).

### 4.4. Static Component Orientation and the Limits of the Anatomic Safe Zone

A central finding was that static acetabular component orientation did not differ between groups. Immediate postoperative CT anteversion (21.8° in the S1-only group vs. 23.1° in the S2AI group; *p* = 0.34) and inclination were similar, and comparable proportions of components in each group fell within the conventional Lewinnek zone. Despite this, the S2AI group had lower spinopelvic mobility, greater femoropelvic reorientation, and lower 1-year HOOS. These findings highlight the limitation of static component orientation alone in characterizing the hip–spine relationship in patients with prior spinal fusion. Previous studies have similarly demonstrated that static component position may not fully reflect functional acetabular orientation during postural change [6,7,22], and reduced standing-to-sitting acetabular reorientation has been described in patients with posterior dislocation [23]. However, functional acetabular orientation during postural change was not directly measured in the present study.

**Figure 4:**
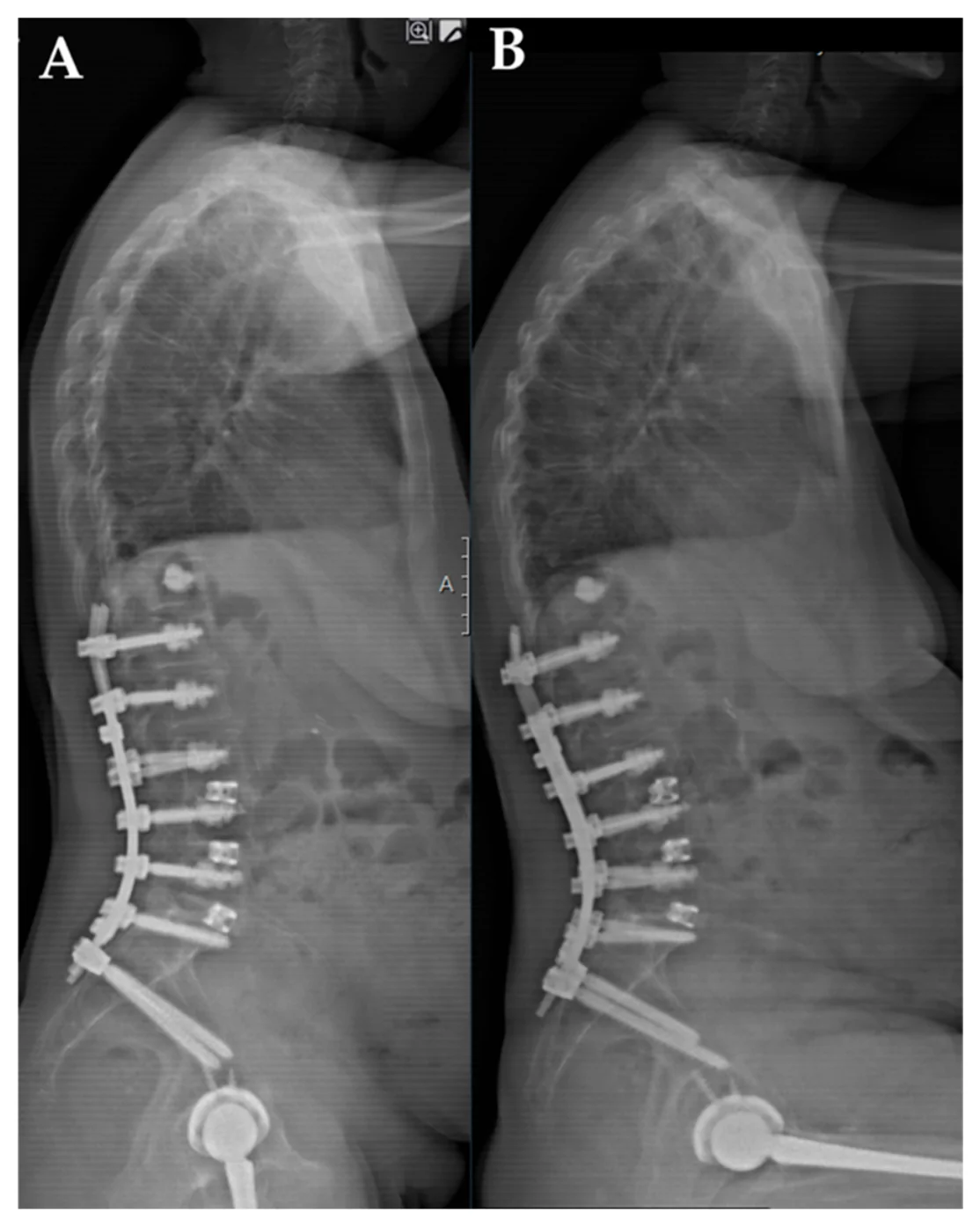
Lateral standing (**A**) and sitting (**B**) EOS radiographs of an S2AI patient who subsequently developed symptomatic anterior impingement. S2AI, S2 alar–iliac; EOS, EOS imaging system.

Four S2AI patients developed symptomatic anterior impingement, and two subsequently underwent revision THA. Both revisions occurred after the standardized 1-year assessment and involved acetabular component revision with head and liner exchange. Although these events occurred despite static cup orientation within the conventional range, the small number of events precludes inference regarding a mechanistic relationship between fixation type, spinopelvic mobility, component orientation, and impingement. More broadly, previous studies have reported increased complication rates and limitations of universal static safe zones in patients with spinopelvic stiffness [24,25,26]. Accordingly, the clinical-event findings in the present cohort should be considered descriptive and hypothesis-generating (Figure 4).

### 4.5. Clinical Implications

These findings have potential implications for the evaluation of patients undergoing THA after prior spinopelvic fixation. Our results highlight the potential importance of considering functional spinopelvic mobility in addition to static component orientation. Standing-and-sitting radiographic assessment and individualized component strategies have been advocated in the literature for patients with spinopelvic stiffness [3,27]. However, the present study evaluated postoperative functional imaging and was not designed to determine whether preoperative standing-and-sitting assessment or individualized cup targeting improves clinical outcomes. Our data also underscore the value of individual rather than group-level evaluation, as group-level differences in spinopelvic mobility and femoropelvic reorientation may not directly predict outcomes in an individual patient.

In patients with spinopelvic stiffness, patient-specific cup positioning and dual-mobility implants have been proposed to reduce the risk of instability [3,27]. However, the present study was not designed to compare these strategies, and the small number of clinical events precludes conclusions regarding their relative effectiveness.

### 4.6. Strengths and Limitations

The main strength of this study is the clear distinction between fixation groups: every S2AI patient had fixation crossing the sacroiliac joint, whereas no S1-only patient did. This allowed standing-to-sitting spinopelvic mobility to be evaluated alongside static CT-based component orientation and a hip-specific patient-reported outcome. A single surgeon and a shared THA protocol reduced variation in surgical technique.

There are several limitations. First, the study was retrospective and not randomized, and the groups were not matched. The S2AI group had longer fusions and greater spinal surgical complexity. Although measured differences were addressed in the multivariable analyses, residual confounding by indication cannot be excluded. The modest sample size also limits the precision of the adjusted estimates and statistical power for rare events such as dislocation and revision, which should be interpreted as descriptive and hypothesis-generating. Given the cohort size and six predictors included in each regression model, the adjusted analyses should be considered exploratory.

Second, ΔSS reflects overall standing-to-sitting pelvic reorientation rather than motion specifically across the sacroiliac joint. Similarly, ΔPFA represents femoropelvic reorientation rather than intrinsic hip range of motion and may be influenced by individual sitting strategy. Femoral orientation in space was not measured directly; ΔPFA is therefore interpreted as the femoropelvic counterpart of ΔSS rather than as an independent measure. In the four staged bilateral cases, the contralateral THA had been performed 6 months before the standardized 1-year EOS of the analyzed hip. Foot support, trunk posture, thigh position, and seat height were not standardized during relaxed-sitting imaging. Static acetabular orientation was measured on postoperative CT and therefore does not capture functional orientation during postural change.

Third, requiring an available standardized 1-year EOS examination may have introduced selection bias, while exclusion of patients who died or underwent subsequent spinal revision before this assessment may have introduced survivorship bias. Observer blinding to fixation type was not feasible because the instrumentation was visible on EOS imaging.

Finally, this was a single-centre study with relatively short follow-up, limiting generalizability and the assessment of late complications. Larger multicentre studies with longer follow-up and better control of spinal surgical complexity are needed to confirm these findings.

## 5. Conclusions

In patients who underwent THA after spinal fusion, S2AI fixation was associated with lower standing-to-sitting spinopelvic mobility and greater femoropelvic reorientation than S1-only fixation. These differences were observed despite similar static CT-based acetabular component orientation between groups. In multivariable analysis, greater ΔSS was associated with better 1-year hip-specific function, whereas fixation type was not independently associated with HOOS after adjustment. Because S2AI fixation was associated with greater spinal surgical complexity and fixation strategy was not randomly assigned, residual confounding cannot be excluded. These findings highlight the potential clinical relevance of residual spinopelvic mobility after THA but do not establish a causal effect of S2AI fixation or a specific preoperative imaging or component-positioning strategy. Larger multicentre studies with longer follow-up and better control of spinal surgical complexity are needed to confirm these findings.

## Figures and Tables

**Table 1:** Baseline demographic and surgical characteristics by group.

Variable	S1-Only (*n* = 30)	S2AI (*n* = 30)	*p* Value
*Demographic characteristics*			
Age at THA, years	67.1 ± 7.8	68.4 ± 7.4	0.51
Female sex, *n* (%)	20 (66.7)	22 (73.3)	0.78
BMI, kg/m^2^	28.1 ± 4.1	28.9 ± 4.4	0.47
*Spinal surgical characteristics*			
History of revision spinal fusion before THA, *n* (%)	5 (16.7)	12 (40.0)	0.08
Number of fused segments	6.6 ± 2.6	8.7 ± 2.1	0.001
Long construct (≥5 segments), *n* (%)	24 (80.0)	27 (90.0)	0.47
UIV at T10 or above, *n* (%)	12 (40.0)	20 (66.7)	0.07
L5–S1 pseudarthrosis/distal failure, *n* (%)	2 (6.7)	7 (23.3)	0.14
PSO/VCR, *n* (%)	3 (10.0)	8 (26.7)	0.18
DXA-confirmed osteoporosis, *n* (%)	7 (23.3)	10 (33.3)	0.57
Fusion-to-THA interval, months	25.6 ± 12.1	27.9 ± 13.5	0.49
*Pre-spinal-fusion sagittal alignment*			
PI, °	54.2 ± 11.1	56.1 ± 11.7	0.52
PT, °	28.1 ± 8.4	31.6 ± 9.1	0.13
SS, °	26.1 ± 9.0	24.5 ± 9.4	0.50
LL, °	36.7 ± 13.2	32.9 ± 14.0	0.28
PI–LL mismatch, °	17.5 ± 13.7	23.2 ± 15.4	0.14
SVA, mm	71 ± 45	91 ± 53	0.12
*Post-spinal-fusion sagittal alignment*			
PT, °	22.0 ± 6.8	23.5 ± 7.1	0.41
SS, °	32.2 ± 7.4	32.6 ± 7.8	0.84
LL, °	49.1 ± 10.7	50.4 ± 11.2	0.65
PI–LL mismatch, °	5.1 ± 8.7	5.7 ± 9.1	0.79
SVA, mm	34 ± 28	38 ± 31	0.60

Values are mean ± SD or *n* (%). Categorical *p* values are from two-sided Fisher’s exact tests. A long construct was defined as fusion involving ≥5 vertebral segments. BMI, body mass index; UIV, upper instrumented vertebra; PSO, pedicle subtraction osteotomy; VCR, vertebral column resection; PI, pelvic incidence; PT, pelvic tilt; SS, sacral slope; LL, lumbar lordosis; SVA, sagittal vertical axis. PI was measured on separate radiographs (after spinal reconstruction vs. 1-year post-THA EOS).

**Table 2:** THA characteristics, static component orientation, and adverse events.

Variable	S1-Only (*n* = 30)	S2AI (*n* = 30)	*p* Value
*THA characteristics*			
Direct lateral approach, *n* (%)	27 (90.0)	28 (93.3)	1.00
Femoral head/bearing type			0.21
28 mm conventional head, *n* (%)	2 (6.7)	0 (0)	
32 mm conventional head, *n* (%)	12 (40.0)	7 (23.3)	
36 mm conventional head, *n* (%)	12 (40.0)	16 (53.3)	
Dual-mobility (32 mm inner head), *n* (%)	4 (13.3)	7 (23.3)	
Dual-mobility vs. conventional bearing, *n* (%)	4 (13.3)	7 (23.3)	0.51
*Immediate postoperative CT (static orientation)*			
Cup anteversion, °	21.8 ± 5.0	23.1 ± 5.4	0.34
Cup inclination, °	41.6 ± 4.8	42.4 ± 5.1	0.53
Anteversion 5–25°, *n* (%)	23 (76.7)	21 (70.0)	0.77
Inclination 30–50°, *n* (%)	28 (93.3)	27 (90.0)	1.00
Both within Lewinnek zone, *n* (%)	22 (73.3)	20 (66.7)	0.78
*THA adverse events*			
Dislocation, *n* (%)	1 (3.3)	1 (3.3)	1.00
Symptomatic impingement, *n* (%)	1 (3.3)	4 (13.3)	0.35
THA revision, *n* (%)	0 (0)	2 (6.7)	0.49

Values are mean ± SD or *n* (%). Dual-mobility constructs (all with a 32 mm inner head) are presented as a separate category and are not included in the conventional femoral head diameter categories. The four-category distribution was compared using the two-sided Fisher–Freeman–Halton exact test; dual-mobility versus conventional bearing was compared using the two-sided Fisher’s exact test. Other categorical comparisons used two-sided Fisher’s exact tests. CT anteversion and inclination represent static supine component orientation referenced to the anterior pelvic plane and should not be interpreted as functional component orientation. Clinical-event comparisons are descriptive because of the small number of events.

**Table 3:** Comparison of standing and sitting spinopelvic parameters, postural changes, and spinopelvic stiffness between the S1-only and S2AI groups.

Parameter	S1-Only (*n* = 30)	S2AI (*n* = 30)	*p* Value
*Standing EOS*			
Sacral slope, °	34.8 ± 7.5	33.1 ± 7.1	0.37
Pelvic tilt, °	21.4 ± 6.8	23.0 ± 7.0	0.37
Lumbar lordosis, °	49.6 ± 10.3	50.1 ± 10.8	0.86
Pelvic–femoral angle, °	183.2 ± 7.9	185.0 ± 8.1	0.39
*Relaxed-sitting EOS*			
Sacral slope, °	16.7 ± 7.3	25.4 ± 6.9	<0.001
Pelvic tilt, °	39.5 ± 8.1	30.8 ± 7.5	<0.001
Lumbar lordosis, °	27.1 ± 11.2	40.3 ± 10.6	<0.001
Pelvic–femoral angle, °	124.5 ± 9.7	116.2 ± 10.1	0.002
*Standing-to-sitting change*			
ΔSS, °	18.1 ± 7.0	7.7 ± 4.8	<0.001
ΔPT, °	−18.1 ± 7.2	−7.8 ± 5.1	<0.001
ΔLL, °	22.5 ± 9.4	9.8 ± 7.0	<0.001
ΔPFA, °	58.7 ± 10.8	68.8 ± 11.7	0.001
Spinopelvic stiffness (ΔSS < 10°), *n* (%)	4 (13.3)	20 (66.7)	<0.001

Values are mean ± SD or *n* (%). All Δ values represent standing-minus-sitting changes. A positive ΔSS indicates a decrease in sacral slope from standing to sitting, whereas a negative ΔPT reflects the increase in pelvic tilt during sitting. ΔPFA represents standing PFA minus sitting PFA and quantifies femoropelvic reorientation during the postural transition. Continuous variables were compared using independent-samples t tests and stiffness distribution using a two-sided Fisher’s exact test.

**Table 4:** Patient-reported outcomes (HOOS and ODI).

Outcome	S1-Only (*n* = 30)	S2AI (*n* = 30)	Between-Group *p*
*HOOS*			
Preoperative, median [IQR]	45 [41–58]	47 [38–59]	0.32
1-year postoperative, median [IQR]	86 [72–92]	79 [68–86]	0.01
Change (ΔHOOS), median [IQR]	+37 [28–40]	+31 [21–39]	0.07
Within-group *p*-value	<0.001	<0.001	—
*ODI*			
Preoperative	38.6 ± 13.8	40.9 ± 14.5	0.53
1-year postoperative	22.1 ± 11.7	24.8 ± 12.5	0.39
Change (ΔODI)	−16.5 ± 12.0	−16.1 ± 13.2	0.90
Within-group *p*-value	<0.001	<0.001	—

Values are presented as median [IQR] for HOOS and mean ± SD for ODI. HOOS was compared between groups using the Mann–Whitney U test and within groups using the Wilcoxon signed-rank test. ODI was compared between groups using independent-samples t tests and within groups using paired t tests. Higher HOOSs indicate better hip-related health, whereas higher ODI scores indicate greater disability. HOOS total score = sum of all 40 items transformed to 0–100 (100 = no symptoms). HOOS, Hip disability and Osteoarthritis Outcome Score; ODI, Oswestry Disability Index. IQR, interquartile range; SD, standard deviation.

**Table 5:** Multivariable linear regression analyses of factors associated with spinopelvic mobility and hip function.

**Model A. Dependent variable: ΔSS (°)**			
**Predictor**	**Adjusted β**	**95% CI**	***p* value**
S2AI fixation (vs. S1-only)	−7.67	−11.30 to −4.02	<0.001
Number of fused segments	−0.50	−1.19 to 0.18	0.16
Revision spinal fusion	−1.80	−5.30 to 1.72	0.31
Preoperative PI–LL mismatch, per 10°	−0.46	−1.62 to 0.70	0.43
Postoperative SVA, per 10 mm	−0.26	−0.90 to 0.38	0.42
Age, per year	−0.09	−0.25 to 0.09	0.29
**Model B. Dependent variable: 1-year HOOS**			
**Predictor**	**Adjusted β**	**95% CI**	***p* value**
Preoperative HOOS, per point	0.28	0.11 to 0.45	0.002
S2AI fixation (vs. S1-only)	−2.10	−6.80 to 2.60	0.37
ΔSS, per 5° greater mobility	3.42	1.21 to 5.62	0.003
CT-based cup anteversion, per 5°	0.80	−1.40 to 3.00	0.47
Number of fused segments	−0.61	−1.54 to 0.32	0.19
Revision spinal fusion	−2.40	−6.90 to 2.10	0.29

Model A: R^2^ = 0.51; adjusted R^2^ = 0.46; overall *p* < 0.001. Model B: R^2^ = 0.46; adjusted R^2^ = 0.40; overall *p* < 0.001. Adjusted β estimates are from multivariable linear regression. Preoperative and postoperative measurements refer to the periods before and after total hip arthroplasty (THA), respectively. Preoperative PI–LL mismatch was measured before THA, whereas postoperative SVA was measured on the 1-year postoperative EOS examination. Because postoperative SVA and ΔSS were assessed at the same 1-year follow-up, Model A estimates represent associations rather than causal effects. Residual confounding by indication and construct complexity cannot be excluded. ΔSS and HOOS were assessed concurrently at the 1-year follow-up; therefore, Model B should not be interpreted as evidence of mediation. ΔSS, standing-to-sitting change in sacral slope; PI–LL, pelvic incidence minus lumbar lordosis; SVA, sagittal vertical axis; HOOS, Hip disability and Osteoarthritis Outcome Score; CT, computed tomography.

## Data Availability

The data presented in this study are available on request from the corresponding author. The data are not publicly available due to ethical and privacy restrictions related to patient confidentiality. During the preparation of this manuscript, the authors used Paperpal for language editing and manuscript formatting assistance. The authors have reviewed and edited the output and take full responsibility for the content of this publication.

## Supplementary Materials

The following supporting information can be downloaded at: https://www.mdpi.com/article/10.3390/jcm15218210/s1; Figure S1: Schematic comparison of distal fixation techniques: S1-only versus S2 alar–iliac (S2AI) fixation. (A) Anterior view of the pelvis with S2AI screw trajectories (blue), which cross the sacroiliac joint into the ilium. (B) Superior (axial) view showing the S2AI trajectories (blue) and S1 pedicle screw trajectories (orange), which terminate within S1. Panels A and B are adapted from *Gray’s Anatomy of the Human Body* (1918). (C) Posterior view of a pelvic model showing the S2AI entry point (red arrow), located between the S1 and S2 posterior sacral foramina. Figure S2: Representative measurements of sacral slope (SS), pelvic tilt (PT), and pelvic–femoral angle (PFA) in standing and relaxed-sitting positions. Table S1: Interobserver and intraobserver reliability of spinopelvic measurements on EOS images (*n* = 60). Table S2: HOOS domain and total scores (0–100; 100 = no symptoms) before THA and 1 year after THA, by fixation group.
