# Supplementary material for: Association of S2 Alar–Iliac Versus S1-Only Fixation with Spinopelvic Mobility and Acetabular Component Orientation in Total Hip Arthroplasty: A Standing–Sitting EOS Study
Source: J Clin Med. 2026 Oct 22;15(21):8210. doi: 10.3390/jcm15218210 (PMC13764318; doi:10.3390/jcm15218210)
Supplement: Supplementary file 1 [file jcm-15-08210-s001.zip › Table S1.pdf]

Table S1. Interobserver and intraobserver reliability of spinopelvic measurements on EOS images (n = 60).

Parameter	Position	Interobserver ICC (95% CI)	Intraobserver ICC (95% CI)
SS	Standing	0.92 (0.90–0.95)	0.95 (0.92–0.97)
SS	Sitting	0.93 (0.91–0.96)	0.94 (0.91–0.96)
PT	Standing	0.95 (0.93–0.97)	0.96 (0.94–0.98)
PT	Sitting	0.92 (0.90–0.95)	0.95 (0.93–0.97)
LL	Standing	0.93 (0.90–0.96)	0.94 (0.93–0.96)
LL	Sitting	0.95 (0.93–0.97)	0.95 (0.91–0.97)
PFA	Standing	0.92 (0.89–0.95)	0.94 (0.91–0.97)
PFA	Sitting	0.93 (0.91–0.96)	0.95 (0.92–0.98)

ICC, intraclass correlation coefficient (two-way random-effects, absolute agreement, single measures); CI, confidence interval; SS, sacral slope; PT, pelvic tilt; LL, lumbar lordosis; PFA, pelvic–femoral angle. Intraobserver measurements were repeated by the first observer after 4 weeks, blinded to the initial results.
