# Supplementary material for: Association of S2 Alar–Iliac Versus S1-Only Fixation with Spinopelvic Mobility and Acetabular Component Orientation in Total Hip Arthroplasty: A Standing–Sitting EOS Study
Source: J Clin Med. 2026 Oct 22;15(21):8210. doi: 10.3390/jcm15218210 (PMC13764318; doi:10.3390/jcm15218210)
Supplement: Supplementary file 1 [file jcm-15-08210-s001.zip › Table S2.pdf]

Table S2. HOOS domain and total scores (0–100; 100 = no symptoms) before THA and 1 year after THA, by fixation group.

	**Pre-THA**				**1 year after THA**			
	**S1-only (n = 30)**		**S2AI (n = 30)**		**S1-only (n = 30)**		**S2AI (n = 30)**	
	**Median**	**IQR**	**Median**	**IQR**	**Median**	**IQR**	**Median**	**IQR**
Symptoms	39	28-56	54	42-64	84	69-94	78	69-88
Pain	47	34-62	42	24-68	86	78-97	84	79-89
ADL	36	22-62	42	38-52	78	73-81	71	65-79
Sport/Rec	35	20-42	42	25-62	69	54-84	62	59-70
QoL	56	45-68	58	42-60	89	79-98	86	81-89
**Total**	**45**	**41–58**	**47**	**38–59**	**86**	**72–92**	**79**	**68–86**

HOOS, Hip disability and Osteoarthritis Outcome Score; THA, total hip arthroplasty; ADL, activities of daily living; Sport/Rec, sport and recreation; QoL, quality of life; IQR, interquartile range (25th–75th percentile). Each domain score was calculated as the sum of its item scores (0–4) divided by the maximum possible sum, multiplied by 100; the total score is the sum of all 40 items on the same scale (100 = no symptoms). Domain scores are descriptive; no between-group testing was performed.
